# Immunization against severe acute respiratory syndrome Coronavirus 2: an overview

**DOI:** 10.4314/ahs.v21i4.11

**Published:** 2021-12

**Authors:** Eman A El-Masry

**Affiliations:** 1 Microbiology and Immunology unit, Department of Pathology, College of Medicine, Jouf University, Al-Jouf, Saudi Arabia

**Keywords:** Breastfeeding, COVID-19, herd immunity, monoclonal antibodies, SARS-CoV, vaccine

## Abstract

In the past years, numerous new fatal infections have emerged, including Ebola, Nipah, and Zika viruses, as well as coronaviruses. Recently, infections with severe acute respiratory syndrome coronavirus 2 (SARS-CoV-2) have emerged in China, and were then transmitted all over the world, causing the coronavirus disease-19 (COVID-19) pandemic, which is transmitted at a higher rate than other diseases caused by coronaviruses. At the time of writing this review, COVID-19 is not contained in most countries in spite of quarantine, physical distancing, and enhanced hygiene measures. In this review, I address different methods for passive and active immunization against this virus, which is known to cause fatal respiratory disease, including natural passive immunization by breast milk, natural active immunization by herd immunization, artificial passive immunization by convalescent plasma or monoclonal antibodies, and artificial active immunization by vaccination. I hope this review will help design a prophylactic approach against outbreaks and pandemics of related coronaviruses in the future.

## Introduction

Coronaviruses can affect many systems in a wide range of hosts^1^. Severe acute respiratory syndrome coronavirus 2 (SARS-CoV-2) belongs to the order Nidovirales, Coronaviridae family, Orthocoronavirinae sub-family, which is sub-divided into four genera: Deltacoronavirus, Gammacoronavirus, Betacoronavirus, and Alphacoronavirus^2^.

The genera Deltacoronavirus and Gammacoronavirus originate from birds and pigs, while Betacoronavirus and Alphacoronavirus genera originate from bats^3^. According to molecular characterization studies, severe acute respiratory syndrome coronavirus (SARS-CoV), Middle East respiratory syndrome coronavirus (MERSCoV), and SARS-CoV-2 were classified as zoonotic Betacoronaviruses2, with 80% nucleotide identity determined for SARS-CoV-2 and SARS-CoV^4^.

Coronaviruses have an outer envelope, a helical nucleocapsid, and a positive-sense, single-stranded, unsegmented RNA genome that encodes 15 non-structural proteins (nsps), eight accessory proteins, and four structural proteins, i.e., spike (S), membrane (M), nucleocapsid (N), and envelope (E) proteins^5^. All these proteins play important roles in the viral replication cycle^6^.

Coronavirus S protein is a multifunctional large trans-membrane protein that projects from the envelope as a trimer, giving the virus its characteristic crown-like appearance^7^. It serves to bind to a specific receptor on the host cell during viral entry at the beginning of an infection^8^. Furthermore, it determines both host range and tissue tropism, and it is a vital immunodominant protein that can induce an immune response of the host^9^.

The ectodomain of the S proteins of all known coronaviruses is divided into two domains (S1 and S2)7. The S1 domain aids binding to the host cell receptor by the receptor-binding domain (RBD), while S2 helps in the fusion process. In SARS-CoV and SARS-CoV-2, the RBD of the S1 protein interacts with the angiotensin-converting enzyme 2 (ACE2) receptor, whereas the RBD of MERS-CoV S1 interacts with the dipeptidyl peptidase-4 (DPP4) receptor. Antibodies targeting this interaction can neutralize the virus and prevent it from entering the cell^10^, ^11^.

The M proteins of coronaviruses are highly diverse in their amino acid compositions, but an overall similarity in structure is preserved among the different genera^12^. They are the most abundant proteins present in the viral particle^13^ and play an important role in viral assembly^14^.

The coronavirus N protein enhances viral transcription, assists the M protein during assembly^15^, and acts as an interferon antagonist^16^.

The E protein of coronaviruses is the smallest among the structural proteins^17^. It is important for viral assembly, release, tropism, and pathogenesis^18^. Moreover, it was shown to act as an ion-channel (viroporin)^19^. Its absence or inactivation may lead to altered virulence^20^.

Coronavirus disease-2019 (COVID-19) is a respiratory condition caused by infection with SARS-CoV-2^21^. It was first detected in Wuhan, China, in late 2019, causing a viral pneumonia outbreak, which was then transmitted all over the world. The World Health Organization (WHO) considered it a pandemic on March 11, 2020. It is primarily transmitted through respiratory droplets. The main symptoms are fever, cough, and shortness of breath. The clinical presentation is mainly mild. Nevertheless, it can present with complications, such as pneumonia and multi-organ failure leading to death, especially in elderly patients and persons with other health problems, such as diabetes or heart disease^22^, ^23^.

At the time of writing this manuscript, COVID-19 is not contained in most countries in spite of quarantine, physical distancing, and enhanced hygiene measures. Due to the absence of approved antiviral drug therapy, we have to depend on two things. The first is implementing infection prevention and control measures to reduce the nosocomial transmission risk^24^. The second is evaluating the different methods for passive and active immunization against this fatal virus, and further improving successful approaches^25^.

Passive immunization involves the transfer of antibodies against a specific organism to a susceptible person for the purpose of preventing or treating an infectious disease caused by that organism. On the other hand, active immunization involves the induction of a variable degree of a specific immune response, which takes variable times to develop depending on the host. Consequently, passive immunization is the preferred method when immediate protection is needed^26^, ^27^.

Generally, passive immunization is more effective as prophylaxis than as a means of treating a disease. When antibodies are used for therapy, they are most effective when taken soon after the beginning of symptoms. The explanation for this temporal variation in efficacy is not well understood, but it is possible that these passively transferred antibodies act by neutralizing the early inoculum that seems to be of a much lesser magnitude than that of well-established disease^28^. Another explanation is that the antibodies passively administered act by modifying the inflammatory response, which seems to be easily done during the early stages of the immune response, while the patient might still be asymptomatic^29^.

## Natural passive immunization by breast milk

Some authors recommend the avoidance of breastfeeding by mothers suffering from COVID-19 as a precautionary principle, without providing strong evidence in support of this recommendation^30^. Based on up-to-date scientific knowledge, SARS-CoV-2 cannot be transmitted through the breast milk of a mother with COVID-19^31^, ^32^.

During the 2002–2003 SARS-CoV epidemic, a pregnant female was infected with SARS-CoV during the second trimester and required mechanical ventilation. After recovery, she delivered a healthy 38-week-old baby. Antibodies against SARS-CoV were detected in the mother's milk and blood around 130 days after the onset of the infection, with no virus detected in the milk and blood^33^.

Similarly, it is speculated that specific SARS-CoV-2 antibodies can pass from a mother with COVID-19 to the baby through her breast milk within few days after she has been infected. These specific antibodies can possibly modulate an eventual SARS-CoV-2 infection of the baby^34^, ^35^.

The WHO and Centers for Disease Control and Prevention (CDC) state that a mother with COVID (suspected, probable, or confirmed) can exclusively breastfeed her baby. She can express her breast milk and feed it to her baby if her general health condition does not allow for breastfeeding. The mother must continuously follow the infection prevention and control measures (droplets and contact precautions), with regular cleaning and disinfection of the surrounding environmental surfaces^32^, ^36^.

With the increasing numbers of confirmed COVID-19 cases, some authors recommend supporting donor milk banking during the COVID-19 pandemic to face the higher demand for donor breast milk than before the onset of the pandemic^37^, ^38^.

All in all, I believe that breastfeeding improves the health of the child, and it is associated with social and economic benefits for the family. Furthermore, it can supply the newborn with protective SARS-CoV-2 antibodies from the mother. At the same time, a mother with COVID-19 must continuously follow the proper infection prevention and control measures.

## Natural active immunization by herd immunization

Induction of herd immunity is a well-tried concept for limiting the spread of a specific disease^39^. Herd immunization denotes the exposure of an animal group or population to an organism to induce an immune response to this organism. Most members in the population will acquire immunity against the inducing organism. Other members that will not develop immunity against this organism are indirectly protected by being safe from acquiring an infection from the protected group^40^.

In addition, during emergencies, recovered and herd-immunized persons can be voluntarily and temporarily recommended for employment in sensitive jobs at airports and hospitals. Such jobs have the possibility of becoming a hot source for spreading the organism. Thus, immune volunteer workers can act as an indirect immunity source to limit disease spread among the target population^39^.

The concept of herd immunization is important during animal husbandry. For example, herds of pigs are exposed to attenuated viruses, subsequently acquire antibodies, and become protected from getting infected, thereby reducing mortality rates^41^.

In view of the dilemma of pandemics in developing and undeveloped countries, where only insufficient numbers of ventilators are available, and most people cannot afford extended lock-down periods, slowing down the spread of the pandemic is of importance to enable the health infrastructure to deal with the consequences. Without an approved vaccine or treatment, some authors recommend making use of localized herd immunity at sensitive locations to limit the spread of the causative infectious agent^42^.

A research of over 20,000 health-care staff in the United Kingdom has found that most people who catch and survive from COVID-19 are likely to be immune for several months afterwards. This study concluded that the immune response acquired after coronavirus infection reduces the risk of catching the virus again by 83% for at least five months 43. In China, it was reported that nearly 14% showed a positive RNA test when re-checked 14 days after complete resolution of COVID-19 symptoms^44^.

Generally, upon herd immunization, it is difficult to reach full immunization coverage; persons with inadequate or missed immunizations should be considered in this scenario^45^. The possibility of viral mutation and emergence of new strains can make herd immunity ineffective^39^.

All in all, I do not trust that full reliance on herd immunity is reasonable without an approved vaccine against SARS-CoV-2, because the elderly will suffer more and more, and the mortality rate will be very high. Moreover, SARS-CoV-2 is not a single entity that may not have a single origin^46^. Anticipating herd immunity using a heterogeneous virus population can be very misleading.

Artificial passive immunization by antibodies

Passive immunization by antibodies is a trusted concept, and was the only method for facing many infections before antimicrobials were discovered^26^, ^27^.

The antibodies used can be laboratory manufactured or isolated from an infected patient's blood. A promising animal study reported a protective effect of passively transferred antibodies from MERS-CoV-immune camels on MERS-CoV-infected mice^47^.

Previous studies reported safe and rapid viral clearance after giving convalescent plasma (CP), especially if given early at the beginning of certain infections, such as MERS-CoV and Ebola virus^48^. Furthermore, this approach has proven effective against infections with H1N1 influenza, H5N1 avian influenza, polio, measles, rabies, and hepatitis B viruses, as well as with SARSCoV^49^–^53^.

Possible sources of SARS-CoV-2 antibodies are CP from COVID-19-recovered persons, monoclonal antibodies (mAbs), or human antibodies induced in genetically engineered animals, such as cows^54^.

Although there are many possible sources of SARS-CoV-2 antibodies, the only source currently available for immediate use is human CP. Passive transfer of antibodies, through CP, collected from COVID-19-recovered persons to protect or treat high-risk individuals is a well-trusted concept. Recently recovered COVID-19 patients that feature high titers of neutralizing antibodies are good sources of CP. The more COVID-19-recovered persons, the more potential donors of CP48,^54^–^59^.

Circulating neutralizing antibodies against different SARS-CoV-2 proteins will be induced in most COVID-19 patients after 2–3 weeks of infection. The CP transferred will provide short- to medium-term humoral immunity against SARS-CoV-2, lasting from weeks to months depending on the amount and composition of the antibodies transferred. The anticipated mechanism of action is mainly viral neutralization. In addition, other possible mechanisms could be antibody-dependent cellular cytotoxicity and/or opsonization^54^.

A single dose of CP containing a high titer of neutralizing antibodies can swiftly decrease viral load and tissue damage with improvement of clinical outcomes, especially if administered early to patients with low viral load or as prophylaxis for highly susceptible persons, including health care workers or family caregivers of COVID-19 patients^55^.

On the other hand, potential risks of CP in COVID-19 need further studies. These risks fall into two groups: first, the risk of transferring any blood products, and second, theoretical risks. The risk of transferring any blood products includes transfer of other infectious agents and induction of immunological reactions, such as serum sickness. The frequency of these risks is low with modern blood banking techniques. However, transfusion-related acute lung injury (TRALI) can occur when using CP therapeutically in patients with lung disease. Therefore, these factors must be considered during risk-benefit assessment, especially in critically ill patients^60^, ^61^. TRALI was reported in an Ebola virus-infected female during treatment with CP^62^.

Theoretical risks comprise the phenomenon of antibody-dependent enhancement (ADE) of viral infections when sub-neutralizing antibody concentrations suppress innate antiviral immunity and enhance intracellular logarithmic viral growth^63^. This special phenomenon was observed in vitro during SARS-CoV infection^64^.

In my opinion, we can use the CP approach while waiting for approval of an effective vaccine or treatment. Further studies are needed regarding optimal dosing of CP, starting time point, definite clinical indications, benefits, and risks, especially in elderly persons.

The use of mAbs is a new era in facing infections, and can be used as bio-therapeutic or passive immunotherapy to fight many viruses. It might be helpful against SARS-CoV-2 with the additional advantage of overcoming several drawbacks associated with serum therapy regarding safety, purity, specificity, and risk of contamination with blood-borne organism^65^.

Numerous reports approved the therapeutic potential of mAbs against many diseases, including various virus-induced fatal diseases^66^^–73^. A cocktail of mAbs can show more effective anti-viral activity regarding prevention and treatment, while avoiding viral escape. This cocktail includes a combination of different mAbs that recognize different viral epitopes, especially during passive immunotherapy^74–76^.

In vitro and in vivo testing of mAbs targeting SARS-CoV and MERS-CoV S proteins showed promising results, and could possibly prove effective against SARS-CoV-2. Examples of neutralizing mAbs targeting SARS-CoV are 80R, CR3014, CR3022, F26G18, F26G19, m396, 1A9, 201, 68, 4D4, and S230. Examples of neutralizing mAbs targeting MERS-CoV are MERS-4, MERS-27, 4C2, m336, G4, D12, JC57-14, MERSGD27, MERS-GD33, LCA60, MCA1, CDC2-C2, 7D10, and G265. Goo et al. reported a set of mAbs targeting six epitopes of MERS-CoV S protein^77^.

Similarity between RBDs of related coronaviruses can result in cross-neutralization. Therefore, SARS-CoV RBD-specific neutralizing mAbs can cross-neutralize the bat SARS-like coronavirus strain WIV1 (RBD with eight amino acid differences to SARS-CoV), but not strain SHC014 (24 amino acid differences)^78^.

SARS-CoV-2 resembles SARS-CoV and MERS-CoV in many genetic, clinical, and epidemiological characteristics. Therefore, SARS-CoV-2 RBD-specific neutralizing mAbs can be identified by comparative analysis of its RBD with that of SARS-CoV, and cross-neutralizing SARS-CoV RBD-specific mAbs could be tested for their efficacy against SARS-CoV-2, and then be assessed clinically^65^.

A certain level of similarity between the RBDs of SARS-CoV and SARS-CoV-2 is mandatory for cross-reactivity to occur. It was reported that SARS-CoV-specific neutralizing mAbs, such as CR3014 and m396, failed to bind SARS-CoV-2 S protein. On the other hand, SARS-CoV-specific neutralizing mAbs, such as CR3022, were found to bind the SARS-CoV-2 RBD^79^.

Cohen reported that the combination of mAbs (anti-SARS-CoV-2 neutralizing mAbs or anti-ACE2 mAbs) and the drug remdesivir is a promising therapeutic option for treatment of SARS-CoV-2^80^. Further evaluation is mandatory before approving this combination therapy.

In my opinion, large-scale production of mAbs is expensive, labor intensive, and time consuming, which balances their clinical applications especially against emerging fatal viruses, such as SARS-CoV-2. Biotechnology companies are battling to produce cocktails of mAbs against SARS-CoV-2, but this is very time-consuming.

## Artificial active immunization by vaccination

There is an urgent need to develop an effective vaccine to prevent future coronavirus epidemics and pandemics. Most successful trials to develop vaccines against previous coronaviruses (SARS-CoV and MERS-CoV) used the respective S protein as a target. The S protein plays a major role in the induction of T-cell and neutralizing antibody responses against these viruses^81, 82^.

Without including the S protein, a trial aiming to induce an immune response by expressing M, N, or E proteins in a recombinant parainfluenza virus type 3 vector (BHPIV3) failed to induce any detectable protection or antibodies against SARS-CoV^83^.

The wide-ranging diversity between different antigenic variants of coronaviruses made the already developed vaccines to have minimal application, even amongst closely related strains of the virus^84^.

Within few months of the SAR-CoV-2 pandemic, multiple pharmaceutical companies started the race to develop an effective vaccine using many platforms, some of which are in the pre-clinical experimental stage^85,86^. The world fights to create an effective and safe Covid-19 vaccine. several vaccines now have been authorized globally and many others remain in the stage of development. Currently, researchers are evaluating 67 vaccines in human clinical trials and 20 have entered the final stages of testing85. At least 89 preclinical vaccinations in animals are being systematically studied. Research institutes and companies are now working toward different vaccines. Most of them targeted towards one of the following three types of vaccines

Life vaccines with vector virus these vector virus vaccines can multiply within humans without causing any diseases. they can be produced also by cell culture.86.

Researchers now are combining one or more genes for SARS-CoV-2 surface proteins. This procedure helps mask some vector viruses, making them “hidden”, as they carry these proteins on their surface, which consequently make them trick the immune system, as if they carry Covid-19 virus. The other vector viruses are not the same as SARS-CoV-2; however, they stimulate producing SARS-CoV-2 proteins in the cells that invaded them. Either way, it contributes to building immune protection that helps to fight a real infection as well. With regard to each case of a vector virus, researchers managed to develop the first dengue vaccine as well as many other experimental vaccines. There are some vector virus vaccines are in development; for instance, Janssen, the German Centre for Infection Research (DZIF), University of Oxford with AstraZeneca, the IAVI / MSD collaboration and the ReiThera / Leukocare / Univer cells collaborate^87,88^.

RNA vaccines contain a specific virus gene in the form of RNA, particularly in the form of RNA, where all living cells engender copies of the individual genes required for the evolution of proteins: messenger RNA (for short mRNA). The mRNA from the vaccine is supposed to induce the evolution of (non-harmful) viral protein in the body after injection, thereby increasing immune protection. Firms as well as institutes that develop such vaccines against Covid-19, including Moderna, BioNTech/Pfizer, Arcturus Therapeutics, CureVac, as well as eTheRNA^89, 90^.

Vaccines Inactivated with Viral Proteins: Such vaccines contain either elected viral proteins (similarly like Novavax, Greffex, University of Queensland, UMN Pharma (subsidiary of Shionogi) and Sanofi / GSK); or include the whole substance for inactivated SARS-CoV-2 viruses (e.g Beijing Institute of Biological Products /Sinopharm). Therefore, it mainly relies on a long-proven technique: multiple confirmed vaccines are manufactured via this method; for instance, vaccines against influenza or hepatitis B or. Nonetheless, it is probably easier to rapidly manufacture larger parts of vaccine units using other vaccines, yet this remains unclear as this will become apparent only when the opportunity arises.^90,91^.

Experiences acquired during SAR-CoV and MERSCoV vaccine development trials denote that SAR-CoV-2 vaccine production is possible after fulfillment and evaluation of the following points: discovery of target antigen(s), route of immunization, animal models, correlated-immune protection, production facility, scalability, outbreak forecasting, target product profile, and target population^92^.

Animal models for SARS-CoV-2 might be difficult to develop, as the virus does not infect wild-type mice, and only causes a mild infection in transgenic animals expressing human ACE2 receptors. To ensure persistent safety and quality of vaccines for human use, they should be produced in processes that comply with up-to-date Good Manufacturing Practice (cGMP). Once satisfactory pre-clinical data are presented, and initial vaccine batches are produced, clinical trials could be started. At first, small phase I trials (to assess vaccine safety in humans) need to be conducted, followed by phase II trials (doses and formulation are established to initially prove efficacy), and then phase III trials (testing safety and efficacy in a larger cohort) can be performed. Finally, vaccines will be available and can be distributed to the global population. However, the demand for vaccines during pandemics might by far exceed the manufacturing capacity^93^,^94^.

The vaccines approved so far are as follows:

Comirnaty (formerly BNT162b2) BioNTech and Pfizer developed this vaccine as a mRNA-modified nucleoside-based vaccine. Fosun Pharma obtained Comirnaty license in China. This vaccine is given as an intramuscular injection with an interval of 21 days, nonetheless, some countries modified this dosing schedule. Comirnaty engenders an immune response against SARS-CoV-2, the virus that induces COVID-19, via encoding a mutated form of the virus's complete spike protein.^95^,^96^

**Moderna COVID 19 Vaccine (previously named mRNA-1273):** Moderna company has developed this vaccine in light of previous studies related to corona viruses. It is a two-dose mRNA vaccine taken 28 days apart. The World Health Organization's Strategic Expert Group (SAGE) on Immunization released guidance on the use of the vaccine in adults.^90,91^

CoronaVac (previously named PiCoVacc) is a formulated alum-enhanced vaccine inactivated with formalin, developed by the China-based biotechnology company Sinovac Biotech.^97^^, 90^

COVID-19 Vaccine AstraZeneca (AZD1222); also called Covishield: AstraZeneca and the Oxford Vaccine Group at the University of Oxford have developed “COVID-19 Vaccine AstraZeneca” (formerly AZD1222 and ChAdOx1), which is a vaccine against the adenovirus chimpanzee. In India, the vaccine is cooperatively developed by the Serum Institute of India and AstraZeneca, and it is called Covishield.^90, 91^

**No name announced**

The research team at Sinopharm and the Wuhan Institute of Virology under the Chinese Academy of Sciences are working to develop an inactive candidate for the COVID-19 vaccine^90, 91^.

**Sputnik V**

The Ministry of Health of the Russian Federation as well as the Gamaleya Research Institute of Russia are working to assess their non-proliferating viral vector vaccine, which is called Sputnik V (previously named Gam-COVID-Vac), in a phase III trial in Russia and globally90, 91.

BBIBP-CorVvaccine

Sinopharm develops second, non-inactivated vaccine for COVID-1998.

**EpiVacCorona**

Biotechnology in Russia (Vector Institute), as well as The State Federal Budgetary Research Institution Research Center for Virology have developed a vaccine named EpiVacCorona, which is a peptide vaccine for COVID-1990.

Covaxin vaccine: Bharat Biotech manufactured a vaccine called Covaxin in collaboration with the Indian National Institute of Virology90.

## Conclusion

The current COVID-19 pandemic can be considered as a reminder of how novel viruses (as SARS-CoV-2) are able to rapidly emerge, spread and cause severe public health crises. Novel prevention and control strategies must be designed to prevent spread of such fatal viruses and reduce their transmission risk to avoid potential future outbreaks and pandemics. Breast-feeding can provide the newborn by protective antibodies from his mother that possibly modulate an eventual SARS-CoV-2 infection in the baby. Anticipating herd immunization by heterogeneous viruses will be very misleading and can increase the mortality rates especially among elderly persons. The use of CP will provide humoral immunity against SARS-CoV-2 lasting from weeks to months so can be used while waiting for approval of an effective vaccine and treatment. The mAbs' cocktail can be tested as bio-therapeutic or passive immunotherapy to fight against SARS-CoV-2 either alone or in combination with anti-SARS-CoV-2 drugs as remdesivir. Several approaches for anti-SARS-CoV-2 vaccine development can be used but to get the prospective vaccine, we should wait at least 6 months after starting clinical trials. There is an urgent need to technology transfer and international collaboration between experts.
